# The Influence of Radial Undersampling Schemes on Compressed Sensing in Cardiac DTI

**DOI:** 10.3390/s18072388

**Published:** 2018-07-23

**Authors:** Jianping Huang, Wenlong Song, Lihui Wang, Yuemin Zhu

**Affiliations:** 1 College of Mechanical and Electrical Engineering, Northeast Forestry University, Haerbin 150040, Heilongjiang, China; wlsong139@126.com; 2 University Lyon, INSA Lyon, CNRS, Inserm, CREATIS UMR 5220, U1206, F-69621 Lyon, France; zhu@creatis.insa-lyon.fr; 3 Key Laboratory of Intelligent Medical Image Analysis and Precise Diagnosis of Guizhou Province, School of Computer Science and Technology, Guizhou University, Guiyang 550025, China; lhwang2@gzu.edu.cn

**Keywords:** compressed sensing, cardiac diffusion tensor imaging, image reconstruction, radial undersampling

## Abstract

Diffusion tensor imaging (DTI) is known to suffer from long acquisition time, which greatly limits its practical and clinical use. Undersampling of k-space data provides an effective way to reduce the amount of data to acquire while maintaining image quality. Radial undersampling is one of the most popular non-Cartesian k-space sampling schemes, since it has relatively lower sensitivity to motion than Cartesian trajectories, and artifacts from linear reconstruction are more noise-like. Therefore, radial imaging is a promising strategy of undersampling to accelerate acquisitions. The purpose of this study is to investigate various radial sampling schemes as well as reconstructions using compressed sensing (CS). In particular, we propose two randomly perturbed radial undersampling schemes: golden-angle and random angle. The proposed methods are compared with existing radial undersampling methods, including uniformity-angle, randomly perturbed uniformity-angle, golden-angle, and random angle. The results on both simulated and real human cardiac diffusion weighted (DW) images show that, for the same amount of k-space data, randomly sampling around a random radial line results in better reconstruction quality for DTI indices, such as fractional anisotropy (FA), mean diffusivities (MD), and that the randomly perturbed golden-angle undersampling yields the best results for cardiac CS-DTI image reconstruction.

## 1. Introduction

To date, almost all clinical magnetic resonance imaging (MRI) is performed by acquiring k-space data along a Cartesian trajectory, which means that data are sampled line-by-line on a rectangular grid. However, k-space can also be sampled in an arbitrary non-Cartesian manner, and different sampling trajectories will have different properties and implications for the reconstructed image [1]. Radial sampling is one of the most frequently used non-Cartesian k-space sampling schemes, firstly proposed by Lauterbur in 1973 [2], which samples k-space along spokes instead of grid lines. Radial acquisitions are very fast and less susceptible to object motion and ghosting artifacts than Cartesian trajectories; it can therefore be significantly undersampled [3,4]. In traditionally uniform radial sampling scheme, k-space is sampled with equally spaced radial lines; each line is restricted to a constant length of the acquisition window and requires a new scan for each desired temporal resolution. The Golden Ratio based profile acquisition scheme was proposed to provide a nearly uniform distribution for an arbitrary number of profiles [5], which was widely applied in MRI, such as cardiac cine MRI [6] and dynamic volumetric MRI [7,8]. Other classical radial sampling strategies include bit-reversed [9] and radial with random angles [10].

Undersampling of radial k-space data provides an effective way to reduce the amount of acquired data while keeping image quality. In radial sampling, the number of sample points is much higher near the center of k-space than the surrounding of k-space. It implies that radial sampling samples low frequencies more densely than high frequencies [11]. However, reconstructions from radial sampling trajectories are more complicated, since they cannot be simply obtained by inverse two-dimensional (2D) Fourier transform. Conventionally, radial acquisition data are reconstructed using projection reconstruction algorithms [2,12,13] or k-space interpolation schemes (e.g., gridding [4,14,15,16,17,18]).

Recently, compressed sensing (CS) appeared as a new mathematical theory for accelerating data acquisitions with high quality from significantly under-sampled data via non-linear reconstruction algorithms [19,20]. An essential factor of CS is incoherent sampling. Pure random undersampling of k-space simplifies the mathematical proofs and particularly guarantees a very high degree of incoherence. However, sampling a completely random subset of k-space is generally impractical due to hardware and physiological considerations [3,21]. Radial sampling that undersampled k-space along spokes can be considered an approximation of a random sampling scheme and also have a variety of significant non-random structures.

The effects of different radial sampling schemes (e.g., uniform-angle, golden-angle, bit-reversed, and random sampling) on the CS reconstruction have been investigated, and the experiments demonstrate that the Golden-angle sampling outperforms the other radial sampling schemes for Breast MRI [9] and for myocardial perfusion MR imaging (MPI) [22]. The combination of compressed sensing, parallel imaging, and radial sampling then provides a fast and flexible way to reduce the amount of acquired data while keeping the integrity of relevant data information. It has been rapidly gaining popularity in different areas of science, and has been proved being able to dramatically improve the quality of undersampled images in MRI [3,4], dynamic MRI [7,23,24], and myocardial perfusion MRI [22,25].

Randomly perturbed uniform radial trajectories increase the incoherence in radial CS-MRI, significantly reduce the streaking artifacts and further improve image quality [26]. The present work aims to investigate various radial sampling schemes as well as the related image reconstruction using CS. In particular, we propose two novel undersampling schemes, namely perturbed radial golden-angle and perturbed radial random angle. The proposed methods are compared with existing radial undersampling methods, including uniformity-angle, randomly perturbed uniformity-angle, golden-angle, and random angle. The experiments are carried out on both simulated and real human cardiac diffusion weighted (DW) images, and reconstruction results are assessed in terms of fractional anisotropy (FA), mean diffusivities (MD), azimuth angle (AA), and elevation angle (EA).

The rest of the paper is organized as follows. Section 2 describes the experimental materials and methods, including datasets description, sampling schemes, and reconstruction methods, and evaluation criteria. Section 3 demonstrates the undersampling scheme’s performance using simulation and real human cardiac datasets; finally, the conclusion is drawn in Section 4.

## 2. Materials and Methods

### 2.1. Sampling Schemes

**Figure 1:**
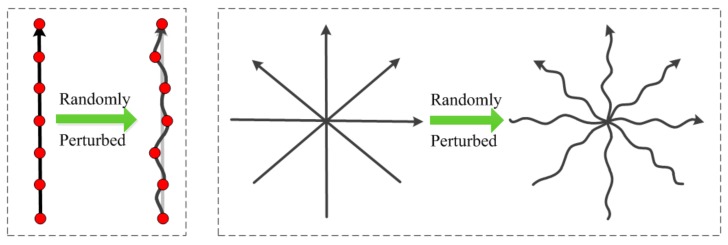
Illustration of randomly perturbed radial lines.

Radial lines are perturbed by adding slight random deviations taken from Gaussian distribution with zero mean and varying variances, as illustrated in Figure 1.

**Figure 2:**
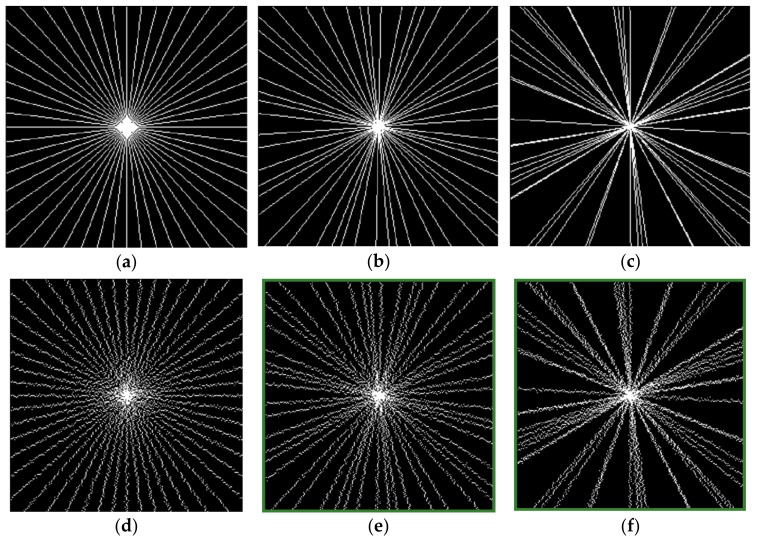
k-space sampling masks in one diffusion direction with sampling rate of 20%. (**a**) Uniform-angle; (**b**) golden-angle; (**c**) random-angle; (**d**–**f**) randomly perturbed sampling schemes corresponding to (**a**–**c**), respectively.

More precisely, six different radial k-space sampling schemes are considered (Figure 2, the sampling ratio in k-space is set to 20%), including uniformity-angle, golden-angle, and random-angle sampling schemes, and the corresponding randomly perturbed ones.

In the uniform-angle sampling, the angle between samples on neighboring spokes (or also called views or profiles) are increased by the constant angle increment $\Delta \phi =\frac{180}{K}$, with *K* indicating the number of spokes. For a set of spokes, this provides the most uniform azimuthal data distribution, as shown in Figure 2a.

In golden-angle sampling, radial projections are successively incremented by the golden angle $\Delta \phi =\frac{\sqrt{5}-1}{2}\cdot 180\approx 111.25^{\circ }$. The golden ratio based profile acquisition scheme was proposed to provide a nearly uniform azimuthal profile distribution in k-space for an arbitrary number of profiles [5]. A relatively well-distributed set is shown in Figure 2b.

In random-angle sampling, spokes are generated from random angles that are uniformly distributed. A reference k-space sampling with sampling ratio of about 20% is shown in Figure 2c, we can see that the randomly placed spokes are clustered in certain regions.

### 2.2. CS Reconstruction

The image reconstruction based on CS from undersampled k-space data consists of solving the following optimization problem:

(1) $$\hat{X}=\arg\;\underset{X}{\min}\left\{\frac{1}{2}{\left\|F^{u}X-Y\right\|}_{2}^{2}+\alpha R\left(X\right)\right\}$$

where, *X* is an image, $F_{l}^{u}=P\cdot F$ is a partial Fourier transform with *F* denoting Fourier transform and *P* the undersampling pattern (mask); *Y* is the undersampled k-space data; R(X) expresses regularization terms that match our prior knowledge of *X*, and makes the highly underdetermined problem in Equation (1) well posed. α is the regularization parameter and satisfies that α>0.

In this work, the undersampled k-space data *Y* of the DW images in all the diffusion gradient directions can be written as:

(2) $$Y=F^{u}\cdot X$$

where $Y=\left[y_{1},y_{2},\ldots ,y_{L}\right]$, $X=\left[x_{1},x_{2},\ldots ,x_{L}\right]$, with columns representing the vectorized DW image, and $F^{u}$ having the following form:

(3) $$F^{u}=\left[\begin{array}{ccc} F_{1}^{u} & & 0\\ & \ddots & \\ 0 & & F_{L}^{u} \end{array}\right]$$

Since the DW images acquired in different diffusion gradient directions have similar anatomical structures, they are somewhat correlated. Consequently, by stacking these images as column vectors of a matrix *X*, the latter will be low rank. Then, the reconstruction of DW images from undersampled k-space data is performed by solving the following optimization problem (i.e., global low rank (GLR) model):

(4) $$\hat{X}=\arg\;\underset{X}{\min}\left\{\frac{1}{2}{\left\|F^{u}X-Y\right\|}_{2}^{2}+\alpha {\left\|X\right\|}_{*}\right\}$$

where ${\left\|X\right\|}_{*}$ is the nuclear norm or sum of singular values of the matrix *X*.

The problem in Equation (4) can be effectively solved using the fast composite splitting algorithm (FCSA) [27]. Let $f\left(X\right)=\frac{1}{2}{\left\|F^{u}X-Y\right\|}_{2}^{2}$, which is a convex and smooth function with the Lipschitz constant *L_f_*, $g\left(X\right)=\alpha {\left\|X\right\|}_{*}$ which is a convex but non-smooth function. Then, the g(X) problem can be solved by a proximal mapping operation [28]: $prox_{\rho }\left(\phi \right)\left(x\right)=\underset{u}{\arg\;\min}\left\{\phi \left(u\right)+\frac{1}{2\rho }{\left\|u-x\right\|}_{2}^{2}\right\}$, where ρ is the inverse of the Lipschitz constant *L_f_*. $\nabla f={\left(\frac{1}{2}{\left\|F^{u}X-Y\right\|}_{2}^{2}\right)}^{\prime }={\left(F^{u}\right)}^{T}\left(F^{u}X-Y\right)$ with ${\left(F^{u}\right)}^{T}$ indicating the inverse partial Fourier transform.

The reconstruction problem for Equation (4) is outlined as in Algorithm 1.
**Algorithm 1.** The solving process of the proposed method.INPUT: K: **the maximum number of iterations;** tol: **the tolerance parameter.** INIT: ρ=1L,t1=1,X0=r1=0,k=0; OUTPUT:  $\hat{X}$: **the reconstructed diffusion weighted (DW) images.****REPEAT:**$$\begin{array}{l} k=k+1;\\ x_{g}=r^{k}-\rho \nabla f\left(r^{k}\right);\\ X^{k}=prox_{\rho }\left(\alpha {\left\|X\right\|}_{*}\right)\left(x_{g}\right);\\ t^{k+1}=\frac{1+\sqrt{1+4{\left(t^{k}\right)}^{2}}}{2};\\ r^{k+1}=X^{k}+\frac{t^{k-1}-1}{t^{k}}\left(X^{k}-X^{k-1}\right); \end{array}$$ UNTIL k>K$\mathrm{OR}\;\frac{{\left\|X^{k-1}-X^{k}\right\|}_{2}}{{\left\|X^{k}\right\|}_{2}}<tol$.

### 2.3. Experimental Data 

**Figure 3:**
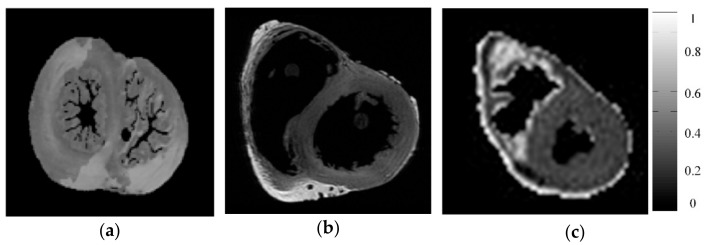
Reference human cardiac DW images. (**a**) Simulated data; (**b**) real data (the first acquisition dataset, the 67th slice); (**c**) real data (the second acquisition dataset, the fourth slice).

The experiments were carried out on both simulated and acquired human cardiac DTI datasets (see Figure 3). Simulated DW images were generated according to the work of Wang et al. [29], which used physical measurements from polarized light imaging (PLI) to generate realistic DW images at different diffusion gradient directions. The simulated DTI data used in this work is obtained with 42 diffusion gradient directions, the b values is 1000 s/mm^2^, and the image size is 128 × 128, as shown in Figure 3a.

Two acquisition datasets for ex vivo hearts were used [30,31,32]. One has been acquired using the following parameters: image size 256 × 256 × 134, image spatial resolution 0.43 × 0.43 × 1.0 mm^3^, and diffusion gradient direction = 21. In the experiment, all the slices (134) were used, for the simplicity, only the B_0_ image of the 67th slice was shown in Figure 3b. This dataset can be downloaded from the website http://cvrgrid.org/data/ex-vivo. The other acquired dataset concerns an ex vivo human heart acquired with a resolution of 2.0 × 2.0 × 2.0 mm^3^, 13 diffusion gradient directions, a bvalue of 1000 s/mm^2^, and the image size of 128 × 128 × 7. To enhance the signal-to-noise ratio (SNR), the acquisitions were repeated six times for averaging. The B_0_ image for fourth slice of this dataset was given in Figure 3c.

### 2.4. Evaluation Criteria

In DTI, diffusion tensor *D* is used to describe the diffusion properties in each voxel, which is a 3 × 3 symmetric and positive definite matrix. There are many measurement indices derived from the diffusion tensor to characterize the water molecule diffusion quantitatively [33,34]. Mean diffusivity (MD) and Fractional anisotropy (FA) are two quantitative parameters commonly used in clinical applications to assess tissue microstructure. They are defined as follows:

(5) $$\mathrm{MD}=\frac{\lambda_{1}+\lambda_{2}+\lambda_{3}}{3}$$

(6) $$\mathrm{FA}=\sqrt{\frac{3\cdot \left[{\left(\lambda_{1}-\mathrm{MD}\right)}^{2}+{\left(\lambda_{2}-\mathrm{MD}\right)}^{2}+{\left(\lambda_{3}-\mathrm{MD}\right)}^{2}\right]}{2\cdot \left(\lambda_{1}^{2}+\lambda_{2}^{2}+\lambda_{3}^{2}\right)}}$$

where, *λ*_1_, *λ*_2_, and *λ*_3_ are eigenvalues of the diffusion tensor D.

In addition, azimuth angle (AA) [35] and elevation angle (EA) [35], as quantitative indices, were calculated from the DW images reconstructed with different methods. The azimuth angle (AA) represents the angle of the fiber in the plane of the section, and the elevation angle (EA) measures the way the fiber escapes from the section [35].

The root of mean square errors (RMSE) between the original indices and the indices derived from the reconstructed images was calculated for each slice in order to quantitatively compare different reconstruction methods:

(7) $$\mathrm{RMSE}=\sqrt{\frac{{\left\|vec\left(x_{rec}\right)-vec\left(x_{ref}\right)\right\|}_{2}^{2}}{N}}$$

where $x_{rec}$ and $x_{ref}$ denote respectively the calculated and the reference indices and *N* is the total number of voxels. Then, the mean RMSE (mRMSE) values of FA, MD, EA, and AA were calculated to evaluate and compare the reconstruction performance.

The regularization parameter α in soft-thresholding operator (in Equation (4)) was set in an adaptive manner using Stein’s unbiased risk estimate (SURE) thresholding [36].

## 3. Results and Discussion

### 3.1. Visual and Quantitative Comparison

**Figure 4:**
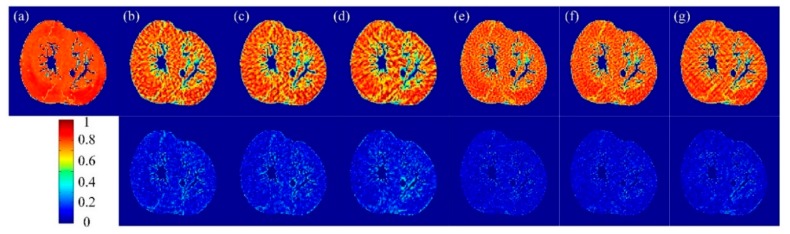
Top row: Fractional anisotropy (FA) maps of the simulated human heart data reconstructed from undersampled k-space data with a 20% sampling rate. Bottom row: FA error maps. (**a**) Reconstructed from the complete k-space data. Reconstructed from undersampled k-space using (**b**) uniform-angle radial; (**c**) golden-angle radial; (**d**) random-angle radial; and (**e**–**g**) the corresponding reconstructions with randomly perturbed radial sampling of (**b**–**d**).

**Figure 5:**
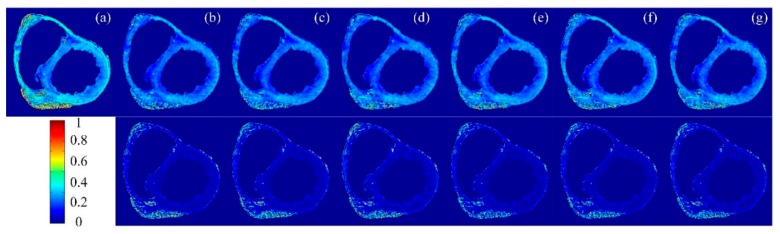
Top row: FA maps of the first acquisition dataset reconstructed from undersampled k-space data with a 20% sampling rate. Bottom row: FA error maps. (**a**) Reconstruction from the complete k-space data. Reconstructions from undersampled k-space using (**b**) uniform-angle radial; (**c**) golden-angle radial; (**d**) random-angle radial; and (**e**–**g**) the corresponding randomly perturbed radial sampling of (**b**–**d**).

**Figure 6:**
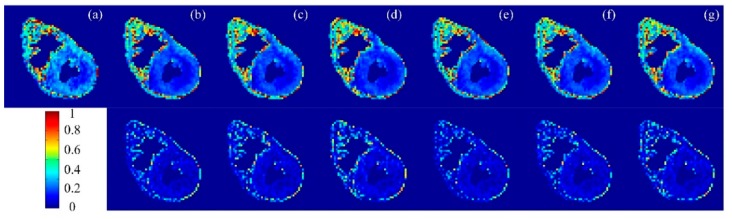
Top row: FA maps of the second acquisition dataset reconstructed from undersampled k-space data with a 20% sampling rate. Bottom row: FA error maps. (**a**) Reconstructed from the complete k-space data. Reconstructed from undersampled k-space using (**b**) uniform-angle radial; (**c**) golden-angle radial; (**d**) random-angle radial; and (**e**–**g**) the corresponding reconstructions with randomly perturbed radial sampling of (**b**–**d**).

**Figure 7:**
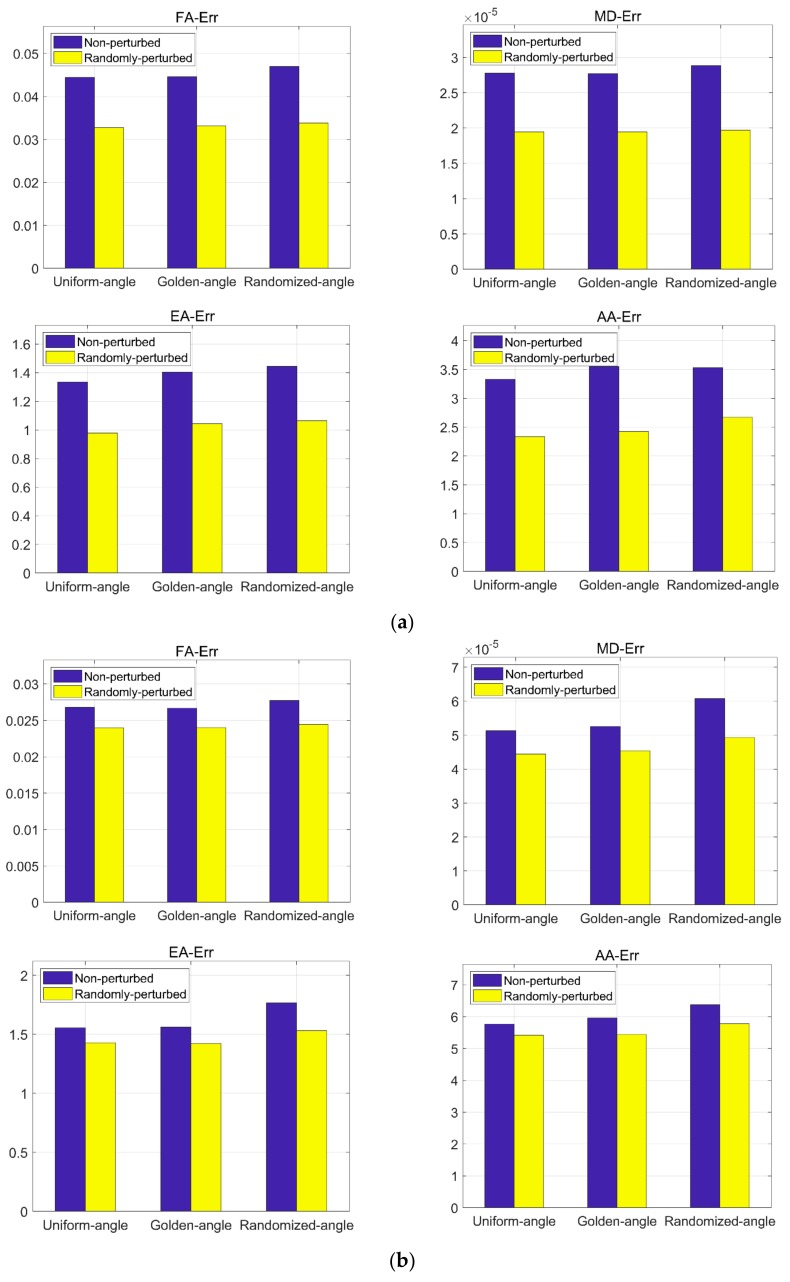

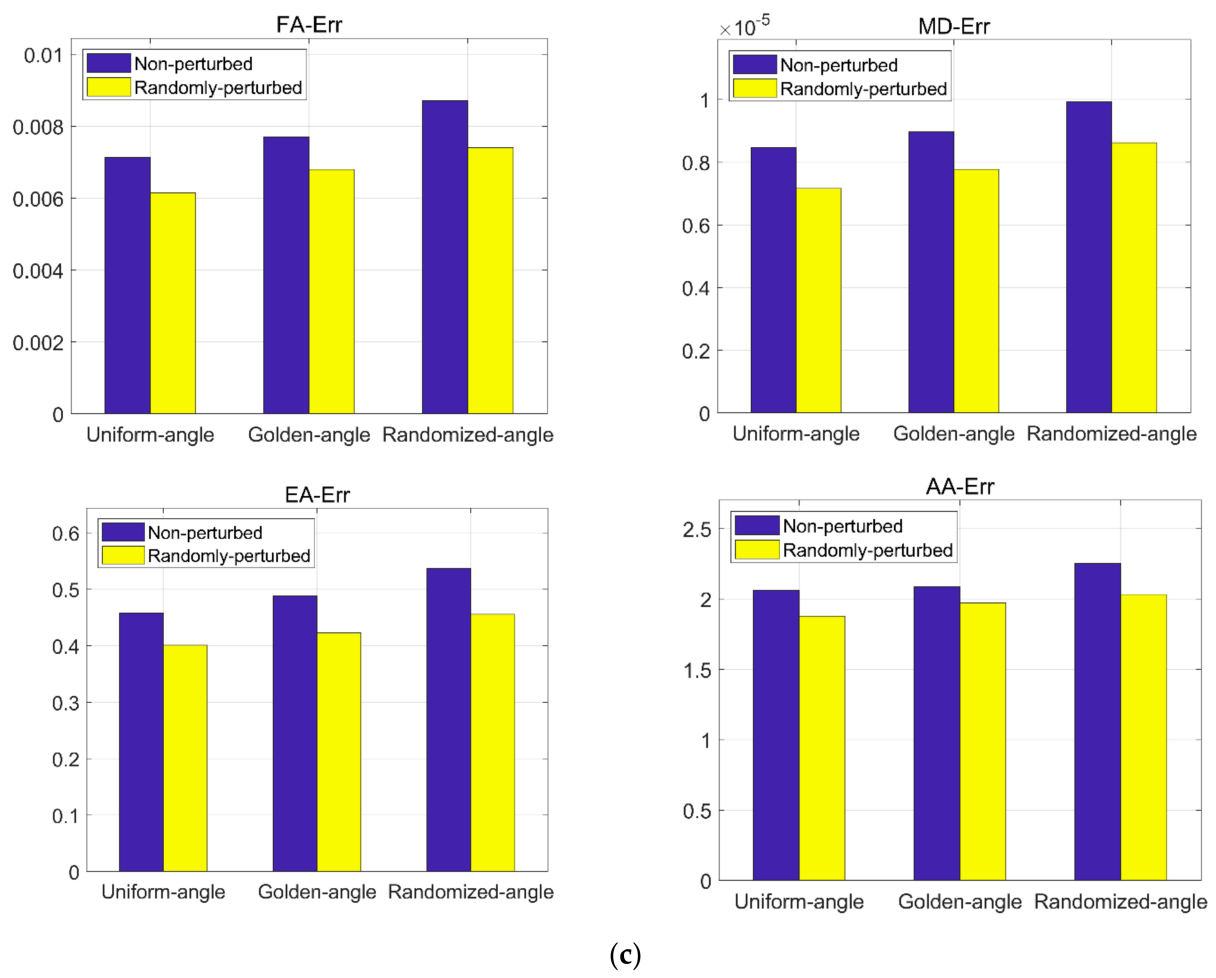
Mean error bars of FA, Mean diffusivity (MD), Elevation angle (EA) and Azimuth angle (AA) for all the datasets. The sampling rate is 20%. (**a**) Mean error bars for the simulation dataset; (**b**) Mean error bars for the first acquisition dataset; (**c**) Mean error bars for the second acquisition dataset.

To illustrate the difference in performance between the proposed methods and the traditional ones, FA maps and the corresponding error maps for three datasets derived from different sampling methods with a sampling rate of 20% were given in Figure 4, Figure 5 and Figure 6. In which, subfigure (a) is the FA reconstructed from the complete k-space data and taken as the reference, subfigures (b)–(d) are FA maps reconstructed from the sampled k-space data with non-perturbed sampling patterns, and (e)–(g) are FA maps reconstructed from the sampled k-space data with perturbed sampling patterns. The second row of these figures represents the error maps. We observe that the randomly perturbed sampling methods proposed in this work were clearly superior to the non-perturbed ones, especially for the simulation dataset (Figure 4) and the second acquisition dataset (Figure 6), but the visual assessment were not evident. Therefore, the visual maps for other indices including MD, EA, and AA were not given in the paper. Instead, the images for the mean of errors were demonstrated in Figure 7.

In Figure 7, the mean error bars for the three datasets used in this work are given, where Figure 7a corresponds to the error bars of FA, MD, EA, and AA for the simulation dataset, and Figure 7b,c represent those for acquisition datasets. The bars filled in purple indicate the mean errors of the reconstructions using the non-perturbed sampling methods, and those filled in yellow indicate the mean errors of the reconstructions using the perturbed sampling methods. Note that the mean error for all the indices using the random perturbed sampling methods are lower than the non-perturbed ones.

To further compare quantitatively these sampling schemes, the mRMSE of all the indices and the corresponding computation times for each iteration for three datasets were given in Table 1. For fair comparison, all the experiments were implemented on a standard personal computer (PC) with 64 bits windows operating system, equipped with a processor of Intel Core i5-2400 (3.10 GHz) and 8GB random access memory (RAM). In Table 1, FA_mRMSE,_ MD_mRMSE,_ EA_mRMSE_, and AA_mRMSE_ represent the mRMSE of FA, MD, EA, and AA respectively, *Itr* means iteration and RP is the abbreviation of randomly perturbed. It can be seen that, for all the datasets used in this work, the mRMSE for all the indices reconstructed from the undersampled k-space data with the proposed sampling schemes are lower than that reconstructed with non-perturbed sampling patterns. Moreover, in terms of mRMSE, we note that the sampling schemes UniformAngleRP and GoldenAngleRP have the best performance.

As to the computation time, for convenience, the computation time for each iteration of the reconstruction using the different sampling schemes was compared. From Table 1, we observe that the computation time of the sampling methods proposed in this work is slightly longer than those non-perturbed ones, but not significant. According to the difference in computation time on the three datasets, we notice that the computation time was more dependent on the size of the image, specifically, the image dimensions and the number of diffusion gradient directions. For example, the size of the second acquisition dataset is 128 × 128 × 13 (13 is the number of diffusion gradient directions), which is the smallest one, and therefore has the fastest computation speed.

### 3.2. Effects of Sampling Rates

**Figure 8:**
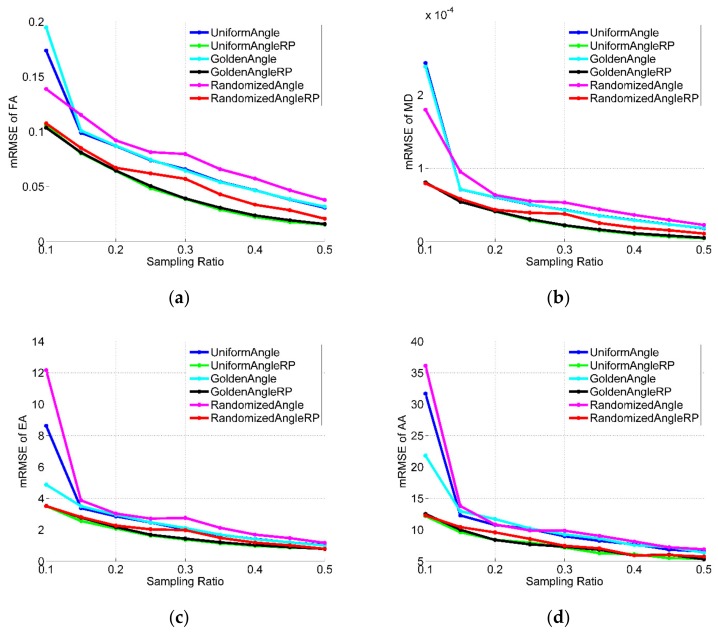
Performance comparisons on simulated data with different sampling rates. (**a**) RMSE of FA; (**b**) RMSE of MD; (**c**) RMSE of EA; (**d**) RMSE of AA.

**Figure 9:**
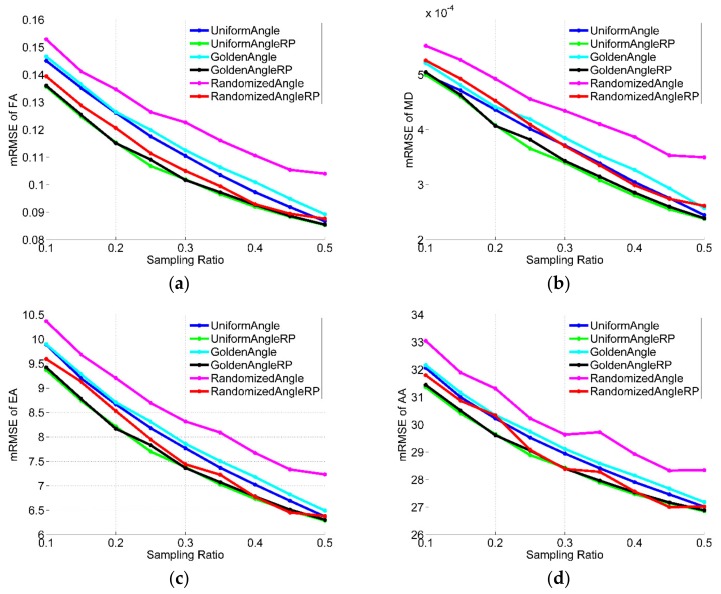
Performance comparisons on the first acquisition dataset with different sampling rates. (**a**) mRMSE of FA; (**b**) mRMSE of MD; (**c**) mRMSE of EA; (**d**) mRMSE of AA.

**Figure 10:**
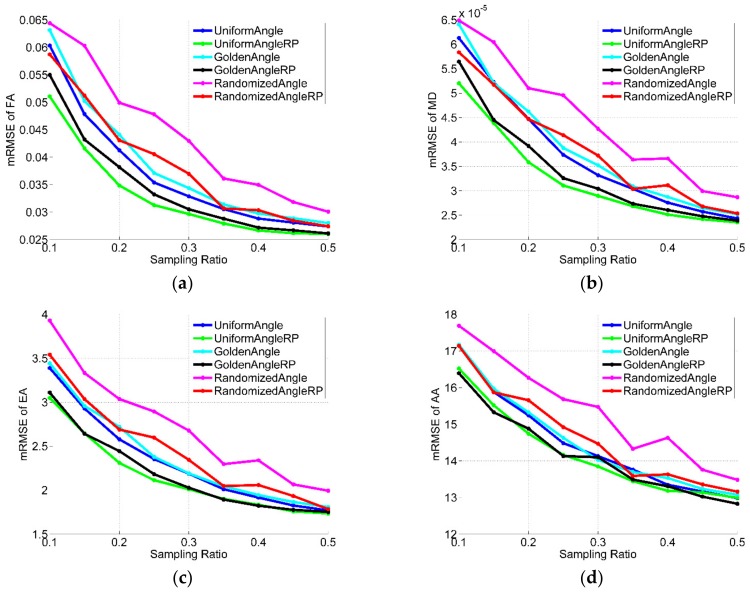
Performance comparisons on the second acquisition dataset with different sampling rates. (**a**) mRMSE of FA; (**b**) mRMSE of MD; (**c**) mRMSE of EA; (**d**) mRMSE of AA.

In the results given above, we used a sampling rate of just 20%. Since different sampling rates will have a great influence on the reconstruction results, the effects of sampling rates for all the datasets were quantitatively analyzed and shown in Figure 8, Figure 9 and Figure 10 (in figures, RP is abbreviation of randomly perturbed). They compares the reconstruction performance, in terms of RMSE of FA, MD, EA and AA, of the different radial sampling makes with various sampling rates of 10~50%. As illustrated in the figures, almost with any sampling rates, the reconstruction from randomly perturbing undersampled radial data resulted in significant reduction in reconstruction error compared to regular radial trajectories. Moreover, the reconstruction error of the uniform-angle and golden-angle radial sampling were almost identical and greater than randomized-angle sampling.

## 4. Conclusions

This work proposed two randomly perturbed radial undersampling schemes for cardiac CS-DTI imaging, namely, randomly perturbed golden-angle and random-angle undersampling schemes. The CS-DTI of human hearts was reconstructed from the sampling data with the conventional undersampling schemes and the proposed ones, and the effects of undersampling schemes on diffusion indices and fiber orientations were compared. The results on both simulation and real datasets demonstrate that the CS-DTI reconstruction with undersampled randomly perturbing schemes resulted in a significant reduction in reconstruction error compared to regular radial trajectories. In addition, the randomly perturbed uniformity-angle and golden-angle radial undersampling schemes are more suitable for cardiac CS-DTI image reconstruction.

## Figures and Tables

**Table 1:** Performance comparisons in terms of root of mean square errors (mRMSE) of FA, MD, EA and AA for different sampling masks on all the datasets with the sampling rates of 20%. (RP is abbreviation of randomly perturbed; the unit of MD_mRMSE_ is ×10^−3^ mm^2^/s).

Dataset	Sampling Scheme	FA_mRMSE_	MD_mRMSE_	EA_mRMSE_	AA_mRMSE_	Time(s)/*Itr*
Simulation dataset	UniformAngle	0.0870	0.0606	2.8677	10.7861	0.3122
	UniformAngleRP	0.0644	0.0413	2.0844	8.4152	0.3218
	GoldenAngle	0.0872	0.0607	2.9675	11.7130	0.3137
	GoldenAngleRP	0.0646	0.0415	2.1862	8.3984	0.3224
	RandomizedAngle	0.0921	0.0636	3.0442	10.8613	0.3104
	RandomizedAngleRP	0.0672	0.0435	2.2745	9.6012	0.3327
The first acquisition dataset	UniformAngle	0.1262	0.4364	8.6682	30.2310	0.5957
	UniformAngleRP	0.1154	0.4085	8.2124	29.6616	0.6011
	GoldenAngle	0.1265	0.4415	8.7086	30.3497	0.5953
	GoldenAngleRP	0.1151	0.4071	8.1684	29.6183	0.6007
	RandomizedAngle	0.1347	0.4928	9.2043	31.3209	0.5963
	RandomizedAngleRP	0.1207	0.4529	8.5320	30.3409	0.6021
The second acquisition dataset	UniformAngle	0.0413	0.0447	2.5788	15.2440	0.0768
	UniformAngleRP	0.0349	0.0359	2.3112	14.7442	0.0788
	GoldenAngle	0.0441	0.0462	2.7211	15.3245	0.0767
	GoldenAngleRP	0.0382	0.0392	2.4450	14.8812	0.0790
	RandomizedAngle	0.0499	0.0510	3.0379	16.2691	0.0766
	RandomizedAngleRP	0.0431	0.0447	2.6901	15.6574	0.0790
